# Autonomic nervous system and inflammation interaction in endometriosis-associated pain

**DOI:** 10.1186/s12974-020-01752-1

**Published:** 2020-03-07

**Authors:** Yajing Wei, Yanchun Liang, Haishan Lin, Yujing Dai, Shuzhong Yao

**Affiliations:** 1 Department of Obstetrics and Gynecology, First Affiliated Hospital of Sun Yat-Sen University, No. 58, the 2nd Zhongshan Road, Yuexiu District, Guangzhou, 510080 Guangdong China; 2 Zhongshan School of Medicine, Sun Yat-Sen University, Guangzhou, 510089 China

**Keywords:** Endometriosis, Autonomic nervous system, Inflammation, Pain

## Abstract

Endometriosis is a chronic inflammatory disease. Pain is the most common symptom in endometriosis. Endometriosis-associated pain is caused by inflammation, and is related to aberrant innervation. Although the specific mechanism between endometriosis-associated pain and the interaction of aberrant innervation and inflammation remains unclear, many studies have confirmed certain correlations between them. In addition, we found that some chronic inflammatory autoimmune diseases (AIDs) such as inflammatory bowel disease (IBD) and rheumatoid arthritis (RA) share similar characteristics: the changes in dysregulation of inflammatory factors as well as the function and innervation of the autonomic nervous system (ANS). The mechanisms underlying the interaction between the ANS and inflammation have provided new advances among these disorders. Therefore, the purpose of this review is to compare the changes in inflammation and ANS in endometriosis, IBD, and RA; and to explore the role and possible mechanism of sympathetic and parasympathetic nerves in endometriosis-associated inflammation by referring to IBD and RA studies to provide some reference for further endometriosis research and treatment.

## Background

Endometriosis is an estrogen-dependent benign gynecological disease. Approximately 10% of reproductive-age women are affected by endometriosis worldwide. This disease is characterized by the presence of ectopic endometrial tissue outside of the uterine cavity [1, 2]. Ectopic endometrial tissues consist of glandular and stromal cells, macrophages, nerves, and blood vessels [3]. Even if the pathogenesis is unclear, endometriosis is certainly a chronic inflammation disorder [4]. The levels and concentrations of active macrophages; interleukin (IL)-1β, IL-6, IL-8; nerve growth factor (NGF); other immune cells; and inflammatory factors are increased in peritoneal fluid (PF) and endometriotic lesions [4–6]. These changes are believed to contribute to serious symptoms of pain such as chronic pelvic pain, dysmenorrhea, and dyspareunia [7]. Notably in deep infiltrating endometriosis (DIE) and intestinal endometriosis, the anatomical distribution of lesions is normally more closely related to pelvic pain symptoms [2]. Abnormal innervations are observed in most endometriotic lesions: an increased number of total intact nerve fibers, increased sensory and decreased sympathetic nerve fiber density (NFD) [6], the occurrence of cholinergic and unmyelinated nerve fibers, etc. [8] In various studies, these abnormal phenomena have been correlated with endometriosis-associated pain [6, 8–10]. More importantly, sympathetic and parasympathetic systems have different inflammation-related effects in different stages of inflammation [10]. Many researchers have found that the function and innervation of the autonomic nervous system (ANS) are altered in chronic inflammatory AIDs [6], such as Crohn’s disease (CD) [11]. However, whether the inflammation induced by abnormal sympathetic and parasympathetic innervation has any effect on endometriotic lesions is unclear. The purpose of this review is to elaborate on the effects of sympathetic and parasympathetic nerve fibers on endometriosis-associated inflammation and to explain the underlying mechanism of action.

## 1. Endometriosis and inflammation

Endometriosis is considered a chronic inflammatory disorder. There are a series of alterations of inflammatory cells, cytokines, and chemokines in endometriotic lesions and PF, forming an inflammatory microenvironment. More importantly, the inflammatory niche also interacts with endometriotic cells (including stromal cells and epithelial cells), which plays an important role in the development and maintenance of endometriosis.

**Fig. 1:**
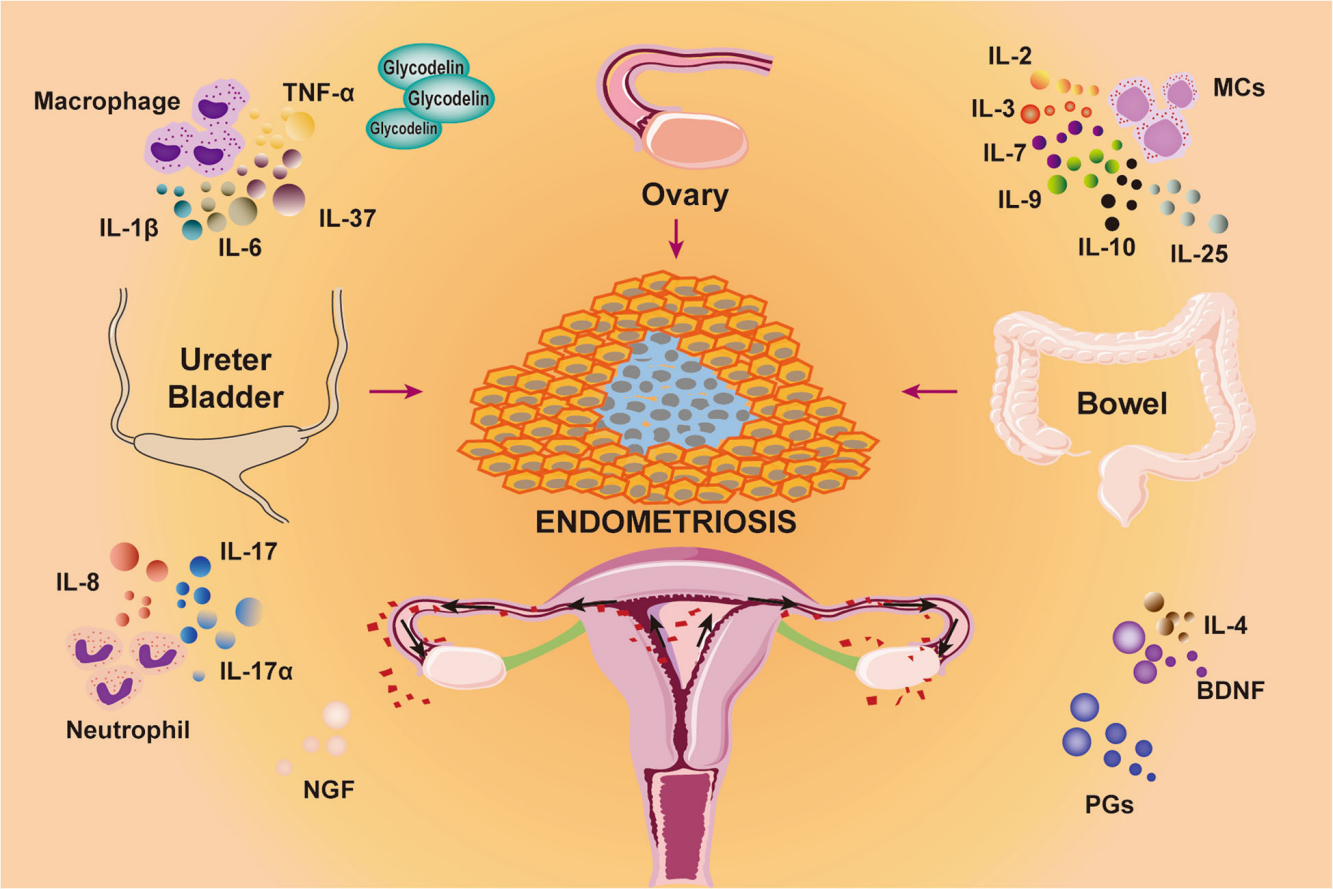
Endometriosis and inflammation menstrual materials retrograde flow to the peritoneal cavity and implant into tissues. Inflammatory cells are recruited to lesions and stimulate multiple inflammatory mediators. These substances form an inflammatory microenvironment and promote the development of lesion that retroact on inflammatory cells and mediators. This vicious cycle contributes to the aggregation of endometriosis-associated inflammation

Menstruation is an inflammatory process characterized by an increase in a variety of tissue-resident immune cells. A complex interaction between resident immune cells and uterine stromal cells modulates the biosynthesis and release of pro-inflammatory cytokines, chemokines, and prostaglandins (PGs), resulting in local vasoconstriction [12]. Menstrual materials retrograde flow to the peritoneal cavity and implant into tissues [13]. During lesion formation, inflammatory cells are recruited to the lesions. The recruited inflammatory cells secrete multiple inflammatory factors. Macrophages secrete and promote the release of IL-1 family factors (including IL-1β, IL-37, etc.), IL-6, and tumor necrosis factor-α (TNF-α) [7, 8, 14]. Mast cells (MCs) release IL-2, IL-3, IL-6, IL-7, IL-9, IL-10, IL-25, and NGF, etc. [5, 7, 10] Neutrophils release IL-8, IL-17, and IL-17α [15–17]. Furthermore, other inflammatory cells secrete factors such as IL-33, MCP1, IL-10, and IL-4. Moreover, endometriotic lesions can induce the expression of PGs, MCP1, glycodelin, and other inflammatory mediators and pain-associated substances [10, 18, 19]. Specifically, PGE2, PGF2α, and TNF-α are produced and increased in the early stage; TNF-α, NGF, and IL-17 can cause persistent inflammation; PGE2, PGF2α, transforming growth factor-β (TGF-β), glycodelin, and TNF-α can induce the sensation of pain [3, 10, 20–22]. These inflammation-associated cytokines, chemokines, other inflammatory mediators, and pain-associated substances act on inflammatory cells in turn. These retroactions lead to more inflammatory cell recruitment in lesions. These substances alter the original environment of peritoneal and pelvic environments and form a new inflammatory microenvironment. The growth, implantation, infiltration, and migration of endometriosis lesions occur subsequently and retroact on inflammatory cells and substances. This vicious cycle contributes to aggregation of the endometriosis-associated inflammation (Fig. 1).

Katherine A Burns et al. suggested that endometriosis is divided into two stages (immune-predominant phase and hormone-predominant phase). They have demonstrated that early stage (< 72 h) of endometriosis is predominantly dependent on the signaling of the innate immune system, whereas estradiol/estrogen receptor α/IL-6(E2/ERα/IL-6)-mediated cross-talk plays a partial role. In this stage of endometriosis animal models, inflammatory factors and vascular endothelial growth factor (VEGF) are increased in mRNA and protein levels, but they are independent of estradiol (E2) treatment. In addition, inflammatory cells are recruited to peritoneal cavity via disease-dependent way regardless of ER. They find that early initiation phase of endometriosis (< 72 h) is largely regulated by the innate immune system. Immune system signaling predominates E2-mediated signaling in disease at this stage. According to their findings, they suggest that there are two phases of endometriosis: immune-predominant phase and hormone-predominant phase. As a result, targeting the innate immune system could prevent lesion attachment in this susceptible population of women [23].

### Inflammatory cells

#### Macrophages

Macrophages, an important member of the immune system, are activated mononuclear phagocytes recruited in endometriotic lesions [14, 24, 25]. Macrophages are classically divided into two typical phenotypes: M1 macrophages (classical activated macrophages) and M2 macrophages (alternatively activated macrophages). M1 macrophages are activated by interferon (IFN)-γ, TNF-α, and lipopolysaccharide. These cells can produce pro-inflammatory cytokines and chemokines that participate in the early stage of injury, pro-inflammatory response, and myoblast proliferation. M2 macrophages are activated by IL-4, IL-10, IL-13, and TGF-β. Once activated, M2 macrophages secrete anti-inflammatory cytokines, growth factors, and other reparative factors, which are involved in the anti-inflammatory response and advanced stage of the repair and healing process [14, 24, 26, 27]. The bidirectional differentiation of total macrophages is caused by different polarizing tendencies of large peritoneal macrophages (LPMs) and small peritoneal macrophages (SPMs). LPMs are involved in an M1 polarization trend and are major contributors to the M1 polarization of total macrophages. Moreover, the tendency of M2 polarization of SPMs plays a major role in M2 differentiation of total macrophages, which appears earlier than the emergence of M1 macrophages [28].

Macrophages are recruited to the peritoneum and lesions of endometriosis. This process usually occurs in the sites of hypoxia and tissue stress, where they clear cell debris, heme-iron, and generate proliferation and pro-angiogenesis signals. In murine, macrophages are required for lesion establishment and growth. Bone marrow-derived Tie-2-expressing macrophages specifically contribute to neovascularization in lesions, possibly because they are associated with the recruitment of circulating endothelial progenitors, and sustain their survival as well as the integrity of the vessel wall [24]. After recruitment to the peritoneal cavity, macrophages mainly present an M2 phenotype, accounting for 80% of lymphocytes in the PF in human endometriosis and mouse endometriosis models [15, 29]. In a mouse model, endogenous macrophages are reportedly involved in tissue remodeling during the development of endometriosis, and the M1 to M2 phenotypic transition is required for the growth of ectopic lesions [30]. Furthermore, transplantation of bone marrow-derived M2 macrophages promotes the development of endometriosis in Balb/C murine models. In the murine endometriosis model, flow cytometry analysis demonstrated that the proportion of LPMs (F4/80^high^CD11b^high^ cells) in the peritoneal cavity decreased immediately with the injection of peritoneal endometrial injection, and the reduced percentage of LPMs persisted until 42 days after injection. It can be proposed that LPMs play a crucial role in the early survival of recurrent endometrial tissues. Then, SPMs participate in complex reciprocities in the pathogenesis of endometriosis [28]. These results suggest that M2 macrophages play an important role in endometriotic lesion establishment and development.

Peritoneal macrophages from patients with endometriosis display increased activation of the pro-inflammatory transcription factor NF-κB and enhanced protein expression of pro-inflammatory cytokines such as TNF-α, IL-6, IL-1β, and TGF-β [1, 5, 8]. NF-κB, a central regulator of gene expression in the immune system, plays a regulatory role in reproductive tissues. IL-6 is under the control of NF-κB. The reduced levels of IL-6 are observed exclusively at the late secretory phase in eutopic endometrium. High serum IL-6 concentrations in endometriosis patients are probably related to the reduction in conserved helix-loop-helix ubiquitous kinase (CHUK) protein as well as NF-κB inhibitor alpha (NFKBIA) mRNA and the p-NFKBIA/NFKBIA protein ratio. Both proteins are involved in the mechanism of NF-κB activation that may affect RELA nuclear location, RELA nuclear/cytoplasmic ratio, and NF-κB DNA binding. The milieus of pro-inflammatory factors create a microenvironment that encourages endometrial cell attachment, invasion, and angiogenesis. In this microenvironment, cell proliferation is enhanced and more inflammatory signaling is activated [31, 32].

Macrophages are abundantly recruited to lesions of endometriosis after activation by certain chemokines and cytokines. These cells can release different types of inflammatory substances, creating an inflammatory microenvironment that contributes to the establishment and growth of endometriotic lesions. In turn, these changes can induce the recruitment of macrophages and as a result, form a vicious circle during the development of endometriosis.

#### Mast cells

In addition to macrophages, the numbers of mast cells (MCs) and activated MCs are clearly increased in human or animal endometriosis [33, 34]. Compared to other sites, DIE lesions have a significantly greater number of MCs, activated MCs and degranulating MCs [8]. MCs are critical sentinel cells in innate and adaptive immune systems. These cells are present in all tissues and are particularly abundant in tissues and mucous membranes that can respond to external inflammatory stimuli and pathogens [35]. MCs are best known for the generation and release of mediators of allergic reactions [35]. Previous studies have indicated a higher prevalence of allergic disease among endometriosis patients. Th2 cells and MCs express IL-25, which is increased in the PF of patients with endometriosis [36]. Moreover, MCs can produce growth factors, costimulatory molecules, and numerous pro- and anti-inflammatory mediators [35, 37, 38]. When activated, MCs release inflammatory mediators from their storage granules as well as via phospholipid membrane metabolism after de novo synthesis of cytokines and chemokines [39]. These mediators include IL-2, IL-3, IL-6, IL-7, IL-9, IL-10, IFN-γ, TNF-α, and chemokines (CXCL8, CCL2, and CCL5) [38]. The activation of MCs is important for proper defense against certain helminth infections and other parasites. However, MCs are potentially lethal cells on widespread activation, which occurs in fatal anaphylaxis, and can cause tissue damage with sustained activation, as in chronic inflammation [35].

RBL2H3 cell (a rat basophilic leukemia cell line, a mucosal mast cell analog) degranulation is increased after E2 treatment [40]. The activation of RBL2H3 cells by E2 triggers the release of biologically active NGF, which promotes neurite outgrowth in PC12 cells and sensitizes dorsal root ganglion cells via the upregulation of Nav1.8 and transient receptor potential cation channel (subfamily V member 1) expression levels. This phenomenon is correlated with endometriosis-related dysmenorrhea [40]. Additionally, activated MCs contribute directly to neuropathic pain symptoms by releasing mediators such as histamine, leukotrienes, tryptase, TNF-α, PGs, serotonin, IL-1, and IL-8. The activation of MCs may also contribute indirectly to the development of neuropathic pain by recruiting leukocytes that release algesic mediators [33]. As mentioned above, IL-25 is expressed by Th2 cells and MCs. The increased level of IL-25 can in turn stimulate IgE production, exacerbating a Th2 response. This process favors the development of allergies by perpetuating a hypersensitivity reaction in endometriosis patients [36].

Both activation and degranulation of MCs have been widely found in endometriotic lesions in animal models and humans. These processes can maintain the state of chronic inflammation and allergic reactions in endometriosis through releasing cytokines and chemokines.

#### Neutrophils

The infiltration of neutrophils into the peritoneal cavity is significantly increased in women with endometriosis compared with that in healthy women, especially in the early stage of endometriosis [23, 41, 42]. In a mouse endometriosis model, neutrophil infiltration in ectopic uterine tissue peaked on days 1–5 and subsequently declined on day 6 or 7, suggesting an important role for neutrophils in the early stages of lesion development [1]. Neutrophils are considered simple foot soldiers of the innate immune system. Neutrophils are undoubtedly major effectors of acute inflammation, and several lines of evidence indicate that they also contribute to chronic inflammatory conditions as well as adaptive immune responses [43]. In endometriosis, neutrophils in the abdominal cavity can secrete an effective pro-angiogenic factor, VEGF, which is also increased in the PF in the endometriosis. As a result, neutrophils may support the growth of endometriosis lesions by secreting VEGF. Moreover, there may be some other nonclassical factors secreted by neutrophils that can promote inflammation and neovascularization in endometriosis [42, 44]. In addition, neutrophils can produce IL-17α which is increased in endometriosis patients and associated with severity and infertility of endometriosis [16, 45–47].

**Fig. 2:**
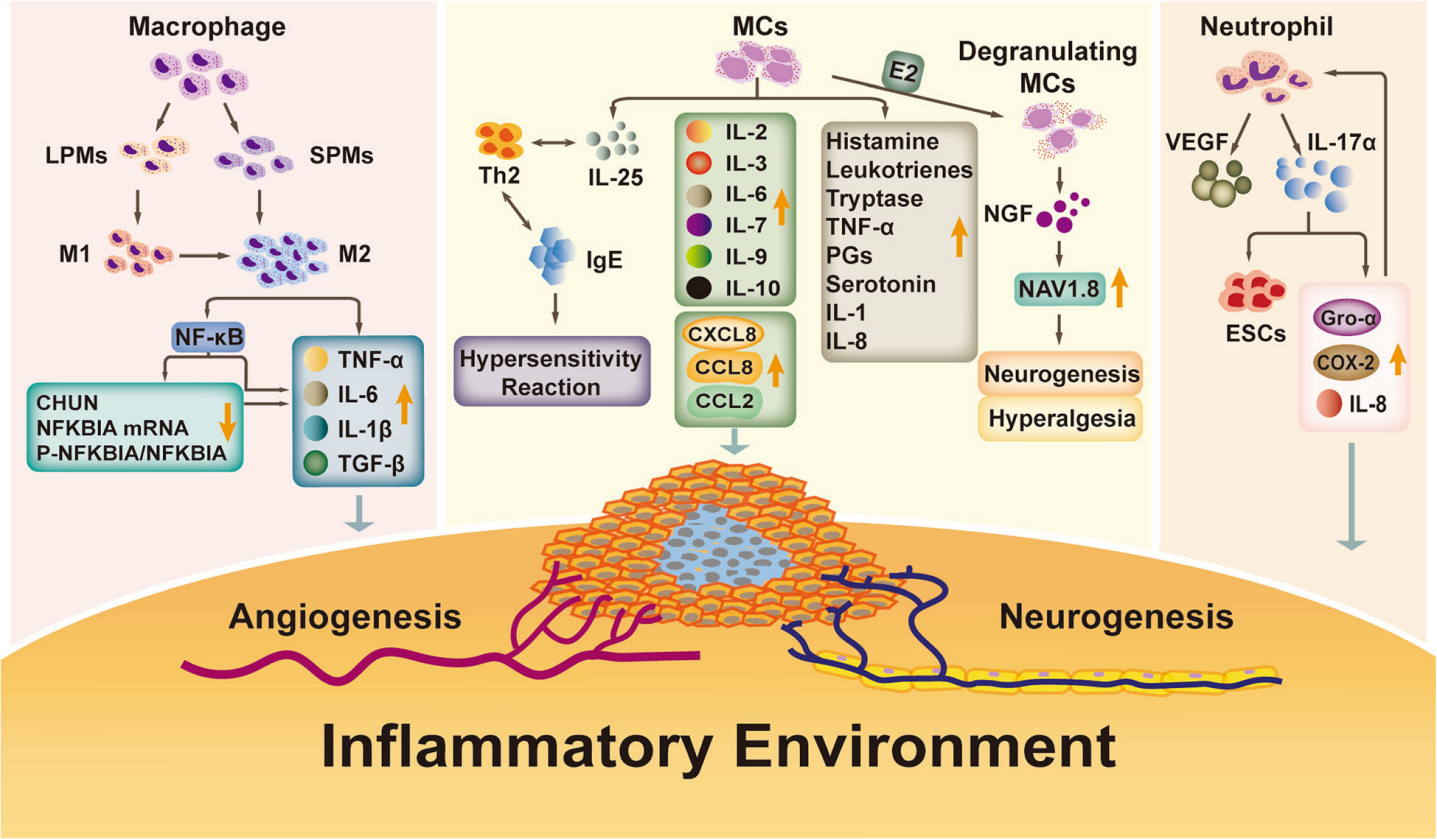
Inflammatory cells in endometriosis macrophages, MCs, and neutrophils are recruited into endometriotic lesions. After macrophage polarization, M2 macrophages secrete multiple cytokines and reduce the expression of CHUN and NFKBIA mRNA and the ratio of P-NFKBIA/NFKBIA. The latter can also promote the secretion of cytokines. Recruited MCs secrete and induce the production of cytokines, chemokines, and other mediators, resulting in angiogenesis, neurogenesis, and hypersensitivity reactions. Under the influence of a high level of E2 (estradiol), MCs degranulate and secrete a large amount of NGF. NGF upregulates NAV1.8, leading to neurogenesis and hyperalgesia. Neutrophils mainly promote the production of VEGF and IL-17α. IL-17α promotes proliferation of endometrial stromal cells (ESCs) and stimulates Gro-α, IL-8, and COX-2 secretion. In turn, the latter can promote the recruitment of neutrophils. These mediators are involved in the formation of the inflammatory microenvironment in endometriotic lesions, causing neurogenesis, angiogenesis and pain

In brief, neutrophils are increased mainly in the early stage of endometriosis and may be correlated with the early development of endometriotic lesions. Neutrophils may affect the occurrence and development of endometriotic lesions through the inflammatory-immune pathway and promote angiogenesis in lesions (Fig. 2).

### Inflammatory mediators

The growth of endometriotic lesions stimulates the production and secretion of pro-inflammatory cytokines, chemokines, and growth factors [3], suggesting that relative inflammatory mediators play a vital role in the development of endometriosis.

#### Interleukins

The largest family of interleukins is the IL-1 family. The IL-1 is a central mediator of innate immunity and inflammation. IL-1 family includes seven ligands with agonist activity (IL-1, IL-1β, IL-18, IL-33, IL-36α, IL36β, and IL36γ), three receptor antagonists (IL-1Rα, IL-36Rα, and IL-38), and an anti-inflammatory cytokine (IL-37) [48]. IL-1 is produced mainly by monocytes and macrophages. The concentration level of IL-1 is increased in the PF, serum, and lesions of endometriosis patients. The imbalance between IL-1α, pro-IL-1β, sIL-1R2, and sIL-1RAcP in the PF and serum of endometriosis patents may be linked to the ability to transform acute inflammation into a chronic one [49]. IL-1β, which reportedly exhibits an excessive concentration and activity in endometriosis, is a potent pro-inflammatory cytokine synthesized by macrophages. IL-1β stimulates the production of eutopic endometrial stromal cells (ESCs) brain-derived neurotrophic factor (BDNF) at the mRNA and protein levels in an IL-1 receptor-dependent fashion by c-Jun N-terminal kinase (JNK), NF-κB, and mechanistic target of rapamycin signal transduction pathways. This process can aggravate endometriosis-associated pain and inflammation [5].

IL-37 is overexpressed in ovarian endometriosis patients [50, 51]. The level of IL-37 reportedly correlates with the severity of endometriosis [52]. IL-37, a novel anti-inflammatory cytokine of the IL-1 family, is generated by various types of immune cells. Normally, IL-37 is present in inflammatory processes and acts as an inhibitor of the inflammatory response. IL-37 binds to IL-18 receptor alpha and delivers the inhibitory signal by binding to TIR8. Moreover, IL-37 can be protective in inflammation and injury as well as inhibit both innate and adaptive immunity [50–53]. This theory can be demonstrated in an endometriosis mouse model [51]. IL-37 synthesis is enhanced by IL-1β, TNF-α, IFN-γ, and TGF-β in endometriosis. However, IL-37 overexpression in turn significantly suppresses both protein and mRNA expression of inflammatory cytokines (IL-1β, IL-6, IL-10, TNF-α, etc.) in cultured endometrial stromal cells. Moreover, a significant inverse correlation was observed between IL-37 mRNA and NF-κB mRNA expression [52]. However, the potential role of IL-37 in the pathogenesis of endometriosis has not been extensively investigated [51]. In other inflammatory AIDs, such as inflammatory bowel disease (IBD), the levels of IL-37 are also increased. Investigations have demonstrated that IL-37 expression could be induced by TNF-α through activation of NF-κB and AP-1. Similarly, IL-37 can be upregulated by cytokines IL-1β and IL-10, reflecting that these cytokines have positive feedback effects on upregulating IL-37 production in inflammatory AIDs. These similar findings suggest a relationship between endometriosis and IBD. The findings of IBD may explain the changes in IL-37 and other inflammatory factors in endometriosis [54, 55].

Other interleukins are also altered in endometriosis patients. The increased IL-8 in the lesions of endometriosis stimulates cell proliferation and has a role in endometriosis as an autocrine regulator of endometrial cell growth. IL-8 has also been implicated as a prominent cytokine involved in angiogenesis [17, 56, 57]. Cell adhesion, transforming growth factor β 1(TGF-β1), and the pro-inflammatory cytokines IL-1β or TNF-α can synergistically promote IL-8 and VEGF expression in ESCs via the p38/ERK1/2 signaling pathways. The high levels of IL-8 and VEGF in the supernatant of ESCs stimulate the angiogenesis of human umbilical vein endothelial cells [58]. In addition, IL-8 exerts chemotactic activity primarily on neutrophils. These roles of IL-8 participate in the pathogenesis of endometriosis and may lead to transformation from acute to chronic inflammation [17, 56]. The levels of IL-25 and IL-17α are also elevated in women with endometriosis. IL-17α, produced by neutrophils [16] or Th17 cells [59], is associated with severity and infertility of endometriosis [16, 45–47]. IL-17α promotes proliferation of ESCs, and stimulates Gro-α, IL-8, and COX2 secretion, recruiting more neutrophils and perpetuating inflammation in endometriosis [16, 47, 60]. The expression of the anti-inflammatory factor IL-10 is enhanced in endometriosis. IL-27 triggers IL-10 production in Th17 cells via c-Maf/RORγt/Blimp-1 signaling to promote the rapid growth and implantation of ectopic lesions [18]. The levels of IL-4 and IL-6 are increased remarkably in women with endometriosis [19, 61, 62]. IL-6 levels are negatively correlated with the total annexin A1 (a pro-resolving and anti-inflammatory mediator) and formyl peptide receptor 2/aspirin-triggered lipoxin expression, suggesting that the reduction in resolution mediators may be responsible for the inflammatory process perpetuation as well as the maintenance and worsening of endometriosis [62].

#### Transforming growth factor β1

TGF-β1 is a pro-inflammatory factor and contributes to inflammatory pain and hyperalgesia [10, 63]. TGF-β1 has been demonstrated to be elevated in the PF of peritoneal endometriosis and DIE. This increased expression is correlated with the severity of dysmenorrhea [8, 10]. TGF-β1 regulates angiogenesis and inflammation in human endometriosis, which in turn provides a favorable microenvironment to attach floating uterine remnants at ectopic sites. TGF-β1 promotes the activity and expression of RHOGTPases (an enzyme that is crucial for cell migration, cytoskeleton dynamics, adhesion, and inflammation) in ectopic endometrial tissues. Aα2-6 sialylation is induced by TGF-β1 through activating TGF-βRI/SMAD2/3 signaling in endometrial cells. In addition, TGF-β1 can activate the P38MAPK molecular target to promote pro-inflammatory cytokines and proteolytic factors. All of the above findings support the important role of TGF-β1 in the pathogenesis of endometriosis [63, 64].

#### Tumor necrosis factor-α

Concentrations of tumor necrosis factor-α (TNF-α) are higher in PF and serum of endometriosis patients, especially in early stages of the disease [36, 65]. Increased TNF-α induces the expression of CXCL16 in eutopic ESCs, which enhances the function of the CXCL16/CXCR6 axis. TNF-α may be associated with the increased motility of eutopic ESCs through the regulation of ERK1/2 signaling. As a result, TNF-α activates systemic and local inflammatory mechanisms in endometriosis development and progression, including elevated levels of chemokines and pro-inflammatory cytokines [66].

#### Prostaglandin

Prostaglandin (PG) is an important mediator of chronic inflammation [20]. The levels of PGE2 and PGF2α are higher in endometriosis and adenomyosis patients and are positively correlated with the severity of vaginal hyperalgesia and dysmenorrhea, respectively [21, 65]. Traditionally, PGs have been thought to function mostly as mediators of acute inflammation. However, recent studies have demonstrated that in addition to their short-lived actions in acute inflammation, PGs can interact with cytokines and amplify the cytokine actions on various types of inflammatory cells, driving pathogenic conversion of these cells by critically regulating their gene expression. One mode of such PG-mediated amplification is to induce the expression of relevant cytokine receptors, which is typically observed in Th1 cell differentiation and Th17 cell expansion. The process of this mode can cause chronic immune inflammation. Another mode of amplification is cooperation of PGs with cytokines at the transcription level to activate NF-κB to induce the expression of inflammation-related genes. This signaling consequently enhances the expression of various NF-κB-induced genes, including chemokines to macrophages and neutrophils, which enables sustained infiltration of these cells and further amplifies chronic inflammation [67].

#### Noninflammatory factors

**Fig. 3:**
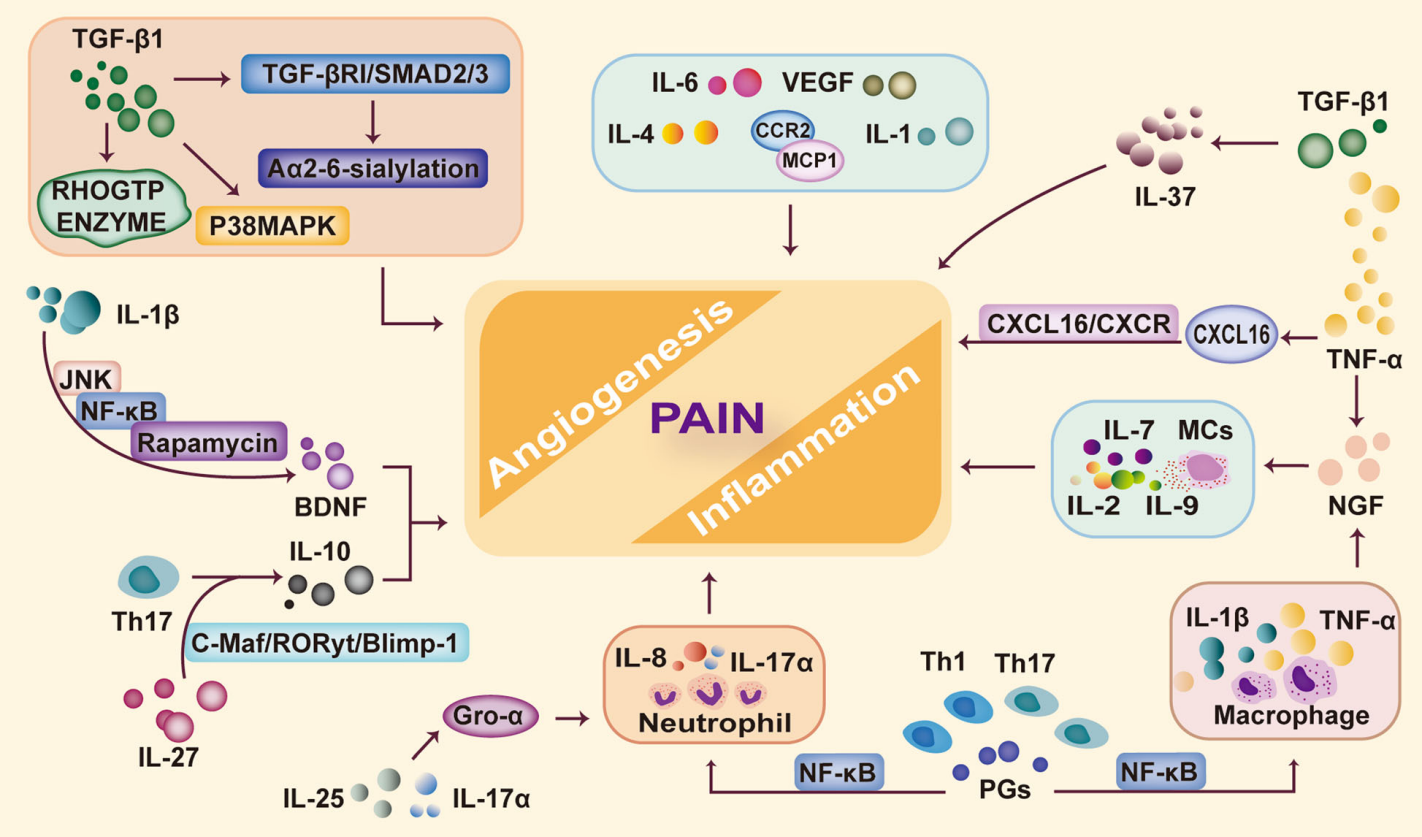
Inflammation-associated mediators in endometriosis abnormal secretion of inflammation-associated mediators can be found in endometriotic lesions. Increased TGF-β1 promotes the activation and expression of RHOGTPases and induces Aα2-6-sialylation through TGF-βRI-SMAD2/3 signaling. TGF-β1 can also activate the P38MAPK molecular target to pro-inflammatory cytokines. IL-1β stimulates the production of BDNF through JNK, NF-κB, and mechanistic target of rapamycin signal transduction pathways. IL-27 promotes IL-10 production in Th17 cells by c-Maf/RORyt/Blimp-1 signaling. IL-25 and IL-17α enhance the secretion of Gro-α to recruit more neutrophils. PGs can also activate NF-κB to promote the expression of inflammation-related genes, and then, more inflammatory cells are recruited (such as neutrophils and macrophages) and stimulate further cytokine release. In addition, NGF, TNF-α, and other inflammation-associated mediators are increased in endometriotic lesions, resulting in the formation of inflammation and angiogenesis and leading to pain feelings in women with endometriosis

In addition to inflammatory factors, noninflammatory factors are associated with endometriosis-associated inflammation in direct or indirect ways. NGF is a neurotrophic factor [68] but is also elevated in lesions of endometriosis. NGF expression is significantly higher in invasive lesions than in noninvasive lesions [22]. Some inflammatory cells are stained by anti-NGF antibodies in the endometriotic interstitium [69]. NGF is upregulated by inflammatory cytokines, such as TNF-α and IL-1β. This neurotrophin is involved in persistent inflammatory pain by activating MC degranulation as well as cytokine production [70]. MCP1, which has a high level in the PF of endometriosis patients, is combined with CCR2 and participates in the process of mononuclear cell infiltration at the site of inflammation [3] (Fig. 3).

## 2. Innervation and endometriosis-associated pain

Pain is the most common symptom in endometriosis patients. This pain may be the result of nociceptive, inflammatory or neuropathic, mechanisms in the form of dysmenorrhea, dyspareunia, or dyschezia [71]. The neurotropic and neuroprotective activity of cytokines suggest that inflammation is one of the major causes of endometriosis-associated pain. Furthermore, anti-inflammatory medication can reduce endometriosis-related pain [72]. The presence of nerves in endometriotic lesions has been confirmed. In humans, murine models and rat models, ectopic endometrium implants develop sympathetic, parasympathetic, and sensory nerve fibers [6, 8, 10, 73]. The ANS, which consists of the sympathetic nervous system and parasympathetic nervous system, was first described in 1916 by John N Langley as an essential mechanism that maintains homeostasis in organisms [74]. The female reproductive system and intestinal tract are imbued with a rich ground plexus of autonomic nerves. In addition to the regulation of vascular and nonvascular smooth muscle contractile activity, intestine movement, glandular secretions, and immune cell interactions, these plexuses can convey information to the central nervous system (CNS) regarding the internal environment and potential noxious stimuli [75, 76]. Changes in the systemic levels of sex hormones (especially estrogen) can regulate the remodeling of uterine sympathetic nerves in nonpregnant females [75, 77]. Many studies have demonstrated that the imbalance among sympathetic, parasympathetic, and sensory innervation and the abnormal secretion of different cytokines can mediate neurogenesis and subsequent peripheral neuroinflammation in endometriosis [26].

### Peritoneal endometriosis

Increased NFD can be detected in peritoneal endometriosis, not in the healthy peritoneum. These nerves mainly consist of Aδ sensory, C sensory, cholinergic, and adrenergic nerves. In particular, sensory nerve density is increased, while sympathetic nerve density is decreased [6, 9, 10]. In addition, Julia Arnold et al. [6] found that PF (collected from endometriosis patients) could induce an increased sprouting of sensory neuritis from dorsal root ganglia (DRG) and decreased neurite outgrowth from sympathetic ganglia (DRG and sympathetic ganglia were dissected from the spinal cord of the Valo specific pathogen-free eggs embryos). They hypothesized that the overexpression of NGF and IL-1β in PF could cause the overbalance of SP-positive nerve fibers, leading to a pro-inflammatory milieu that in turn affected PF in a vicious cycle [6]. Nerve fibers are located in or near endometriotic stromal cells and are colocalized with immature blood vessels. Furthermore, sympathetic nerve density is significantly higher in the adjacent tissue of lesions [78]. Infiltration of retroperitoneal endometriosis follows the pelvic pathways, while parasympathetic nerves are occasionally involved in infiltration. It is not clear why that happens because sympathetic nerves are present everywhere in the body, whereas endometriosis just occurs in pelvis preferentially rather than in all parts of the body [79].

The pelvic nerve emanates from the inferior hypogastric plexus and supplies sympathetic and parasympathetic innervation to the pelvic viscera. The inferior hypogastric plexus’s efferent fibers reach to rectum, uterus, rectovaginal septa ventrally, and finally the deep vesicouterine ligament [80, 81]. The vagus nerve provides parasympathetic nerve fibers to the entire abdomen proximal to a point approximated by the splenic flexure of the colon [82]. There is a complex environment in the pelvic and peritoneal cavity due to the anatomic structure and function of each visceral organ. Altered innervation in endometriosis lesions leads to changes in pelvic cavity innervation. Complex neural networks may transmit the pain produced by lesions to the brain.

### Ovarian endometriosis

The human and mammalian ovaries are innervated by sympathetic, sensory, and a few parasympathetic nerves. Nerve fibers in ovarian endometriosis are a mixture of sensory, sympathetic, and parasympathetic nerve fibers. The ovary receives sympathetic innervation from the upper lumbar spinal segments via splanchnic nerve fibers and parasympathetic innervation via the vagus nerves. The autonomic axons reach the ovary through the ovarian nerve plexus and the superior ovarian nerve [2, 75, 83]. The study of Ricu M and colleagues showed that sympathetic nerves are involved in the control of early follicular growth [83]. The nerves of the fallopian tube basically regulate the smooth muscle contractility and ciliary beat activity of the fallopian tube [84]. The sympathetic innervation in fallopian tubes is regional variations. In the ampulla, nerves are known to be associated with blood vessels. The innervation of the isthmus is much denser and participates in neural control of the sphincter [75]. Parasympathetic nerves are relatively less dense in the fallopian tube and are mainly confined to the vasculature and muscular layer [75, 84].

Brett et al. [72] found a direct association between endometriosis-associated nerve fibers in peritoneal lesions and significantly higher menstrual pain. Ovarian endometriosis patients who reported the lowest pain were most unlikely to have endometriotic lesions with associated nerve fibers [72]. However, another study demonstrated multiple sympathetic and sensory nerves in ovarian endometriosis with a particularly high density (nerves/mm^2^) of the sympathetic nerves [85]. Compared with women without endometriosis, PGP9.5 (a highly specific pan-neuronal marker), NPY (a marker of sympathetic nerve fibers), and VIP (a marker of parasympathetic nerve fibers) positive nerve fibers are decreased in the isthmus of the oviduct of endometriosis patients [84].

It can be assumed that when the ovarian innervation of endometriosis patients changes, ovarian electrophysiological activities as well as follicular activities are affected. In addition to the ovary, nerve fibers in the fallopian tube isthmus of endometriosis patients are decreased, leading to abnormal release of neurotransmitters. Subsequently, the smooth muscle contractility and ciliary beat activity of the fallopian tube are affected. Due to these various factors, normal female ovarian function is changed. The dysfunction of ovary and fallopian tube may lead to infertility in some women affected by endometriosis.

### Deep infiltrating endometriosis

Hyperalgesia, a major characteristic of “neuropathic pain,” and severe chronic pelvic pain are most commonly found in patients with deep infiltrating endometriosis (DIE) nodules [8, 86]. DIE usually develops in anatomical sites with rich innervation. Typical sites are the rectovaginal septum, pararectal space, uterosacral ligament, rectum, ureters, diaphragm, and other less common sites [8, 87]. Sympathetic and sensory nerves are widely distributed in these areas under physiological conditions [11]. Some researchers have reported that immune-expression of NPY (sympathetic fibers) and VIP (parasympathetic fibers) are greatly higher in the uterosacral ligament and adjacent connective tissue in women with DIE. Transection of the uterosacral ligament to block these fibers can relieve pelvic pain [2, 88, 89]. There is no difference in NFD between lesions of the cul de sac, uterosacral ligament, and pelvic sidewall [8]. Other studies reported the loss of sympathetic nerve fibers in/near the lesions of intestinal endometriosis, especially in the mucosal and muscular layers. However, the density of sensory nerve fibers was unaltered in all of the analyzed areas near the endometriotic lesions [2, 11]. NFD is highest in rectal lesions compared with that in other sites of DIE and six times higher than that in normal rectal walls. Pathological examination found that these nerve fibers usually invade ESCs. Polyclonal rabbit anti-human vesicular acetylcholine transporter (VAChT), which is a specific marker for cholinergic fibers, can be present in parasympathetic neurons. The VAChT enzyme is richly expressed in nerve fibers of rectal lesions but weak or absent in other endometriotic lesions [8]. Furthermore, GAP-43, a marker for neurite outgrowth, is selectively expressed in neurons associated with intestinal DIE lesions [3].

The enteric nervous system is vital for many gastrointestinal physiologic functions. The gut microbiota regulates the adult enteric nervous system via a major neuronal growth factor 5-hydroxytryptamine. 5-Hydroxytryptamine acts as a promoter of intestinal mucosa growth and also as a suppressor of inflammation in intestinal mucosa [90]. The human gut wall is richly innervated by sympathetic nerve fibers and by sensory fibers expressing the neuropeptide substance P (SP). In a mouse model, enteroendocrine cells (i.e., neuropod cells) synapse with vagal neurons to transduce gut luminal signals in milliseconds by using glutamate as a neurotransmitter to the brainstem [76]. Muscularis macrophages (MMs), which reside between the circular and longitudinal muscle layer of the bowel wall, are closely related to enteric neurons in the myenteric plexus. CSF-1 produced by enteric neurons modulates the inflammatory response of neighboring MMs [91].

As mentioned above, NFD in intestinal endometriosis is six times higher than that in the normal intestinal wall [8]. We can assume that neurogenesis is increased as endometriosis-related lesions develop. Due to the influence of various factors, the production of CSF-1 is enhanced after macrophage recruitment, contributing to the survival of macrophages [91]. MMs can regulate the adjacent inflammatory response [91]. Neuropod cells not only connect to the synapses of innate vagus nerve fibers but also connect to the synapses of new vagus nerve fibers and parasympathetic nerve fibers [76]. With the aggravation of endometriosis-associated inflammation, changes in the gut microbiota and inflammatory microenvironment [90], and the increased concentration of PG [21, 65], endometriosis-associated pain become more severe. These factors may contribute to greater pain. Next, the pain signal is transmitted to the brain through the interaction of neuropod cells and synapses [76]. At length, it may cause women with DIE (especially with intestinal endometriosis) to experience more pain sensation than women with other types of endometriosis.

### Neurotrophins and endometriosis-associated pain

Endometriosis-associated pain is related not only to the number and distribution of nerves but also to some neurotrophins, especially BDNF and NGF.

In addition to the inflammation-associated changes in NGF mentioned above, the following changes of NGF are correlated with nerve fibers and neuropathic pain. NGF is a vital mediator of pain and inflammation [6]. NGF/TrkA signaling, implicated in neurodegenerative disorders (including Alzheimer’s disease), chronic pain, inflammation, and cancer, plays a key role in neuronal development, growth, survival, and function [92]. NGF induces the expression of SP and calcitonin gene-related peptide, which are neuropeptides involved in modulation of central pain transmission [65]. Moreover, NGF promotes the sprouting of nociceptors, increases the number of sensory neurons, and contributes to persistent inflammatory pain [65]. In endometriosis, NGF expression is significantly higher in invasive lesions than in noninvasive lesions [22]. Inflammatory cells stained with an anti-NGF antibody in the endometriotic interstitium are commonly observed in human endometriosis and rat endometriosis models [69]. The immunointensity of NGF and its receptor TrkA is markedly elevated in the endometriotic epithelium and stroma of women with DIE. NGF immunointensity in the stroma is also significantly associated with local nerve bundle density and deep dyspareunia intensity [3, 93] Furthermore, suppression of NGF via siRNA in a surgically induced rat model of endometriosis inhibits both endometriotic lesion growth and NFD and reduces hyperalgesia [3]. These results suggest that abnormal innervation in endometriosis is associated with endometriosis-associated inflammation.

BDNF, another kind of neurotrophin, can promote cell growth, survival, and differentiation in several classes of neurons [68]. Moreover, nociceptor-derived BDNF, in addition to participation in the process of inflammation, has effects on promoting peripheral and CNS damaging pain [94]. BDNF is an important factor mediating the transition from acute to chronic pain [95]. The concentrations of BDNF in plasma and PF are significantly greater in women with endometriosis than in women without endometriosis. The expression levels of BDNF mRNA in ectopic lesions are markedly increased compared with those in eutopic and control endometrium. These results are correlated with endometriosis pain. IL-1β can stimulate the production of BDNF via an IL-1 receptor-dependent fashion at the mRNA and protein levels. This effect of IL-1β is mediated by JNK, NF-κB, and mechanistic target of rapamycin signal transduction pathways [67, 96]. BDNF immunostaining is identified in adjacent sections, predominantly localized in the stromal and granular compartment of the DIE lesion, but not in the surrounding muscle tissue. In addition, immunohistochemistry revealed the in situ colocalization of macrophages, BDNF, and nerve fibers in endometriosis lesions [5]. These results suggest that BDNF plays a role in inflammatory pain and neuropathic pain through some signaling pathways. Moreover, when assessed for their ability to distinguish between women with revised Classification of the American Society of Reproductive Medicine stage 1 and 2 or 3 and 4 disease and controls, BDNF was the only presumptive marker capable of identifying stage 1 and 2 disease [97]. This finding suggests that plasma BDNF is a potentially useful clinical marker of endometriosis superior to CRP, CA-125, NT4/5 and NGF.

## 3. ANS in other chronic inflammatory disorders

The sympathetic nervous system (SNS) and parasympathetic nervous system (PNS) are classically balanced for maintaining homeostasis. It has been proven that ANS is disrupted in the process of various chronic inflammatory diseases (irritable bowel syndrome, IBD, RA, etc.), and the structure and function of the ANS are altered [10, 11, 98, 99].

The vagus nerve system is the major component of PNS, and it has a dual anti-inflammatory role through its afferent and efferent fibers [100]. SNS exhibits a more complex bidirectional influence. There are evidences that the loss of intestinal sympathetic innervation elicits an innate immune-driven inflammation [101]. In addition, the loss of sympathetic innervation is the hallmark of various acute and chronic inflammatory processes (such as colitis, arthritis, insulitis, etc.) in inflammatory lesions [102–104]. The SNS and vagus nerve have synergistic reaction via the splenic nerve to inhibit the release of TNF-α by macrophages of the peripheral tissues and the spleen [98]. As a result of the decreased concentration of sympathetic anti-inflammatory neurotransmitters, an inflammatory-promoting microenvironment is formed and triggers the following pain-generating signal pathway [99].

However, the anti-inflammatory role of SNS is controversial. The activation of the SNS can strengthen or suppress the activity of immune cells [105, 106]. The reaction of immune cells to neurotransmitters is alterable depending upon the context of receptor engagement, such as microenvironment, cytokine milieu, activation state of cells, and expression pattern of neurotransmitter [106]. On the systemic level, the SNS has pro-inflammatory effects during the initial/early stage while anti-inflammatory effects in the later stages (e.g., AIDs and collagen-induced arthritis) [6, 10, 106]. Moreover, the imbalance of the ANS can be a predictor of various neuro-immune disorders. In particular, autonomic dysfunction, represented by low parasympathetic activity, precedes the development of some chronic inflammatory disorders, such as rheumatoid arthritis (RA). This finding suggests that autonomic dysfunction could be the etiopathogenesis of inflammatory disorders rather than a consequence of chronic inflammation [98].

There are many similarities between certain inflammatory disorders and endometriosis. Both IBD and endometriosis are chronic inflammatory disorders. The differential diagnosis between IBD and endometriosis is important in women with abdominal pain [107]. Simone Ferrero et al. demonstrated that sympathetic innervation of mucosa and the muscular layer around colorectal endometriotic lesions are significantly decreased [11]. These results are similar to those of CD [102], which suggests that similar changes in nerve fibers may be involved in the inflammatory response in CD and endometriosis [11]. Besides IBD, there are also many similarities between RA (a chronic inflammatory disorder) and endometriosis. The level of PGE2 is increased in synovial fluid of RA patients [108] and in PF of endometriosis women [21, 65]. Similar to endometriotic PF and lesions, the concentration of M2 macrophages is increased in synovial tissues [15, 29, 30, 109]. In addition, sympathetic nerve fibers are decreased and BDNF-positive cells are increased in RA synovium [110]. These findings suggest that RA may have something in common with endometriosis in pathogenesis. The following sections will briefly describe the mechanism of ANS changes in IBD and RA.

### Inflammatory bowel disease

Inflammatory bowel disease (IBD), a chronic recurrent gastrointestinal tract disease, is divided into ulcerative colitis (UC) and Crohn’s disease (CD) [100, 111]. The pathophysiology of IBD includes genetic, immunological, and environmental factors. IBD is characterized by production of various pro-inflammatory cytokines, such as TNF-α and IL-1β, and ANS dysfunction [100, 112]. The intestine is dominated by the ANS, which is composed of the sympathetic and parasympathetic nervous systems. Sympathetic and parasympathetic nerve fibers provide afferent information from the gut to the brain and inform the CNS about the intestinal microenvironment [113, 114]. It has been proven that emotional or psychological disorders and autonomic dysfunction can alter visceral sensitivity, usually by lowering the pain threshold [115, 116]. The most common is stress, which can alter the brain-gut axis, leading to a wide range of gastrointestinal disorders, including IBD, irritable bowel syndrome, and other functional gastrointestinal diseases [117, 118] Moreover, stress acts as a vital factor in changing the ANS and can cause long-term modifications in sympathetic and vagus nerve balance [119]. Stress inhibits the vagus nerve and stimulates the SNS via autonomic-related projection neurons of the PVH to the dorsal motor nucleus of the vagus nerve and sympathetic preganglionic neurons of the spinal cord. Because of the anti-inflammatory properties of the vagus nerve through its afferent and efferent fibers, stress has pro-inflammatory properties [120].

The sympathetic dysfunction in CD and vagal dysfunction in UC have been reported [102, 113, 121]. The decreased sympathetic innervation in the intestinal wall of CD patients gives rise to the predominance of pro-inflammatory SP-positive nerve fibers compared with tyrosine hydroxylase (TH)-positive nerve endings [11, 102]. Sympathetic nerve fibers are increased in UC patients [113]. Other studies have also shown that the level of catecholamine (CA) increases in UC patients, while it decreases in CD patients [121].

The specific mechanism of the difference of changes of sympathetic nerves between CD and UC is still not clear. However, more and more researchers agree that the SNS plays either pro- or anti-inflammatory role depending on the time point of SNS modulation [106, 122]. In the beginning of early inflammatory response, neurotransmitters (such as norepinephrine (NE)) of the SNS support chemotaxis and extravasation of leucocytes. At the same time, the concentration of NE can affect the metabolic state of macrophages. After leucocytes encountered in an inflammatory process, they begin to produce and secrete proinflammatory factors and sympathetic nerve repellent factors. Then release of neurotransmitters is inhibited and loss of sympathetic nerves occurs [101, 122]. This may be the reason of alteration of sympathetic nerve fibers density in lesions of some inflammatory disorders in different periods.

It is generally believed that β-adrenergic receptor (β-AR) inhibits many immune cells of innate immune system (such as neutrophils, macrophages, etc.). Low concentration of NE can enhance TNF level of macrophage via α-2-AR-mediated action or β-AR signaling [123]. High and low concentration of NE binds to β-AR and α-AR respectively. In addition, NE can stimulate Th2 immune responses by increasing IL-4, IL-6, and IL-10 through β-AR signaling. On the contrary, Th1 immune responses (such as production of lymphocyte TNF, IL-2, and IFN-γ) are suppressed by β-AR signaling [122]. These results suggest that high NE might promote immune diseases with predominance of Th2 cytokines (such as UC), whereas low level of NE can activate innate immune system in chronic symptomatic phase (macrophage, neutrophils, etc.) of CD which is Th1 lymphocyte dominant [122]. The Th1 cells express high levels of β2-AR, whereas expression is undetectable in Th2 cells [121]. Spleen is innervated by adrenergic pathways. The vagus nerve anti-inflammatory activity relies on ChAT-expressing T cells or B cells in the spleen and requires an intact splenic nerve (splenic nerve is one kind of sympathetic nerve) [124]. After activated of adrenergic splenic nerve, it can release NE into spleen activating memory T lymphocytes which produce acetylcholine (ACh) [125]. Stimulated release of ACh from T cells occurs following activation of β2-AR by NE [126], and then, downstream responses are activated. UC can cause hyposplenism or compromise spleen function, placing these individuals at risk for developing “overwhelming post-splenectomy infections” [125]. Besides, the increased sympathetic nerve in UC lesions may also affect expression of β2-AR. It may explain the mechanism of pathological sympathetic changes in UC. However, different environments of receptor engagement lead to altered responses of immune cells to neurotransmitters, and SNS can either enhance or inhibit the activity of cells associated with the acquired/adaptive immune system [105, 106]. Consequently, it may explain the different role of the SNS in pathogenesis between UC and CD.

In particular, CD has more similarities to DIE in terms of symptoms, changes in the number of nerves in the lesion, and other aspects. Both CD and endometriosis affect the bowel and can cause abdominal pain. Ileal endometriosis is a differential diagnosis of CD because both can cause inflammation, induration, thickening, and stricturing of the small bowel [127]. Sympathetic nerve fibers are decreased in all layers in CD patients [102]. Macrophages and fibroblasts in inflammatory lesions produce nerve repellent factors specific for sympathetic nerve fibers, such as Semaphorin 3C (Sema3C). Sema3C-positive crypts in the mucosa of CD are significantly increased and are negatively related to the density of mucosal sympathetic nerve fibers [102]. In addition, the reduction in parasympathetic tone can be found [115]. These nerve changes are similar to the DIE mentioned above [2, 11].

**Fig. 4:**
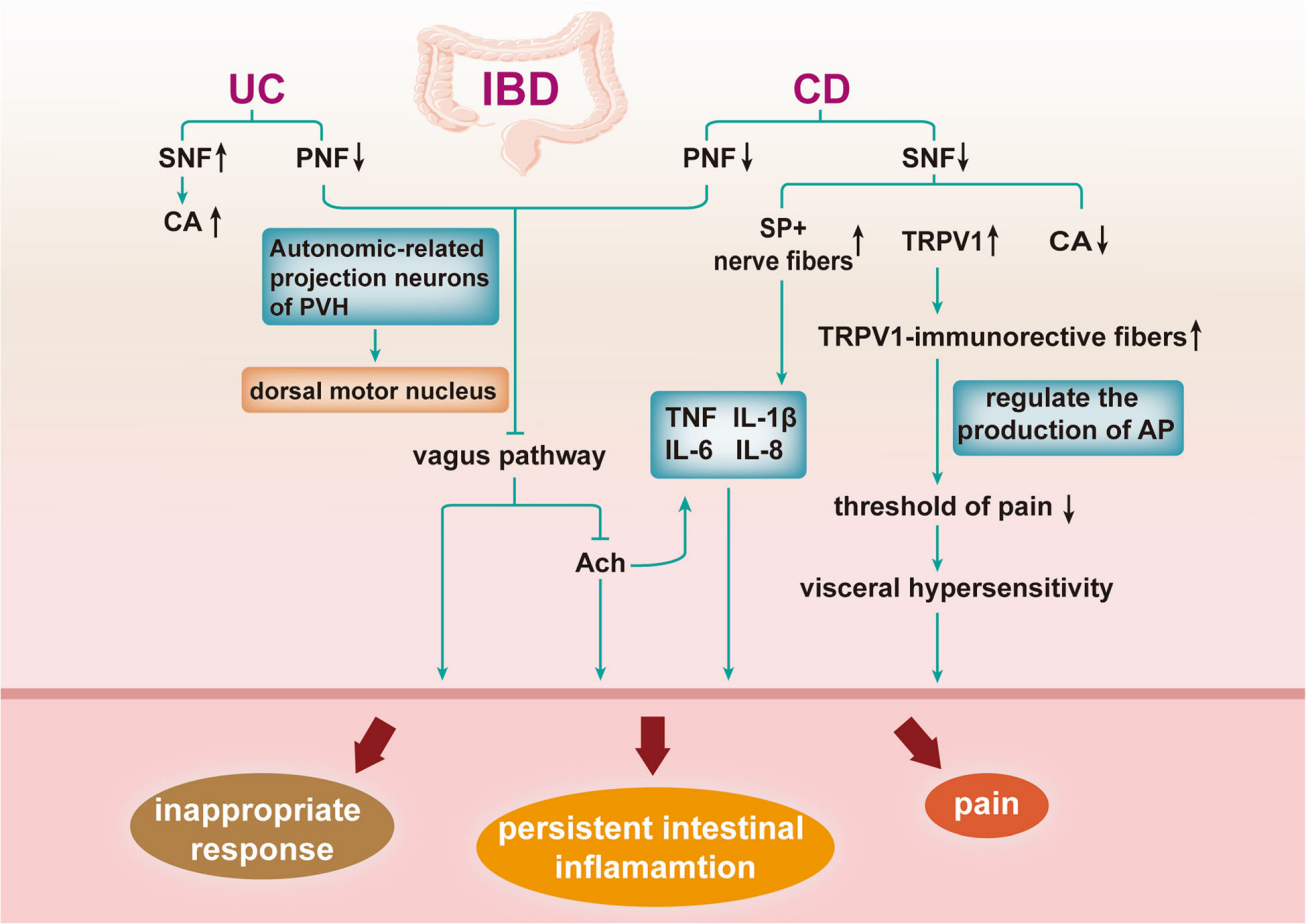
Relevant mechanisms of IBD Sympathetic nerve fibers (SNFs) are increased in UC, leading to an enhanced concentration of CA. Parasympathetic nerve fibers (PNFs) are decreased in UC and CD, resulting in inhibition of the vagus pathway through autonomic-related projection neurons of the PVH to the dorsal motor nucleus of the vagus nerve. This inhibition of the vagus pathway suppresses the secretion of ACh, which promotes increased cytokines and contributes to persistent intestinal inflammation as well as inappropriate responses. Decreased PNFs also occur in CD. Reduction in SNFs in CD lead to increased SP-positive nerve fibers, elevated TRPV1, and reduced CA concentrations. The former promotes the secretion of TNF, IL-1β, IL-6, and IL-8. Elevated TRPV1 contributes to the enhancement of TRPV1-immunorective fibers, which can reduce the threshold of pain via regulation of action potentials production. These processes can enhance visceral hypersensitivity. These factors can work on IBD lesions, form a persistent intestinal inflammatory environment and make patients feel pain

The loss of sympathetic nerves results in a decrease in the concentration of anti-inflammatory neurotransmitters. The neuropeptide SP plays a role in pro-inflammatory effects by inducing the secretion of pro-inflammatory cytokines, such as TNF, IL-6, and IL-8 [10, 102]. The vagal reflex pathway (the cholinergic anti-inflammatory pathway) has prominent effects on the regulation of the inflammatory response. The vagus nerves can perceive signals from the microbiota and then bring this intestinal information to the CNS, where they can integrate the information to produce the adaptive or inappropriate response. The latter can maintain the pathological condition of the digestive tract or be propitious to the development of disorders. The cholinergic anti-inflammatory pathway is mediated by synapses on enteric neurons that release ACh at the synaptic junction with macrophages through vagal efferent fibers. ACh binds to the α-7-nicotinic ACh receptors of macrophages to inhibit the release in TNF. Accordingly, the decrease of parasympathetic tone in CD patients reduces the stimulation α-7-nicotinic ACh receptors, which leads to an increase in cytokines such as TNF and consequently to persistent intestinal inflammation [115, 128]. The histochemistry results of neural tissue of the excised end of ileum have shown that transient receptor potential cation channel subfamily V member 1 (TRPV1) is increased. Multivariate regression analysis suggests that TRPV1-immunorective fibers and MCs are correlated with abdominal pain score, proposing that these fibers might lead to visceral hypersensitivity [129]. The accumulation of CD4^+^ T cells, induced by chronic inflammation in the mouse colon, and endogenous opioids has an effect on analgesia. This effect at least in part works through modulation of voltage-gated ion channels underlying action potential electrogenesis and TRPV1 [129, 130] (Fig. 4).

### Rheumatoid arthritis

Rheumatoid arthritis (RA) has many common features with endometriosis, such as inflammation and autonomic dysfunction. RA is a chronic inflammatory disease. The concentrations of macrophages and cytokines (such as TNF) are increased [109, 131]. Nerve repellent factors of sympathetic nerve fibers Sema3C and Sema3F can be found in the lesions of RA [103]. In addition to inflammation, autonomic dysfunction exists. Autonomic dysfunction occurs before the development of RA, and there is a causal relationship between autonomic nervous dysfunction and the development and progression of RA. This finding could be relevant for endometriosis and other immune-mediated inflammatory diseases. In the RA patients, the activity of PNS is reduced, whereas the SNS is overactive. In RA synovium, researchers can find a low density of sympathetic nerve fibers. Moreover, the level of NE is lower in RA patients than in arthritis patients [110, 132–134]. The depletion of CA before the onset of arthritis in an RA animal model plays an anti-inflammatory role, suggesting that CA might promote the immune response before the onset of disease. The lower level of NE could lead to the activation of α-AR types. Activation of α-AR may lead to a more pro-inflammatory status, but little information is available [132, 134]. In addition to the activation of α-AR, β2-AR is reduced in the mouse model of RA—collagen-induced arthritis (CIA) [135]. It has been proven that sympathetic neurotransmitter NE inhibits the inflammation of CIA by suppressing the differentiation and function of Th17 cells through β2-AR signaling. Th17 cells can produce specific cytokines (such as IL-17 and IL-22), leading to pro-inflammatory reactions. β2-AR, produced by CD4^+^ T cells, is downregulated in CIA. NE activation of β2-AR reduces the transition from CIA-induced CD4^+^ T cells to the Th17 phenotype. This activated β2-AR-cAMP-PKA signaling in Th17 cells undermines the CIA-induced cell inflammatory response to exert the anti-inflammatory effect [135]. Furthermore, when the concentration of NE gets higher, NE binds to β2-AR and transmits the information through GalphaS (Gαs)-coupled proteins. However, this anti-inflammatory pathway can be disrupted in an inflammatory environment. The Gαs-pathway (high cyclic AMP) of receptor coupling can switch to the Galphai (Gαi)-pathway (low cyclic AMP) in RA mixed synovial cells, which is a pro-inflammatory signal [103].

**Fig. 5:**
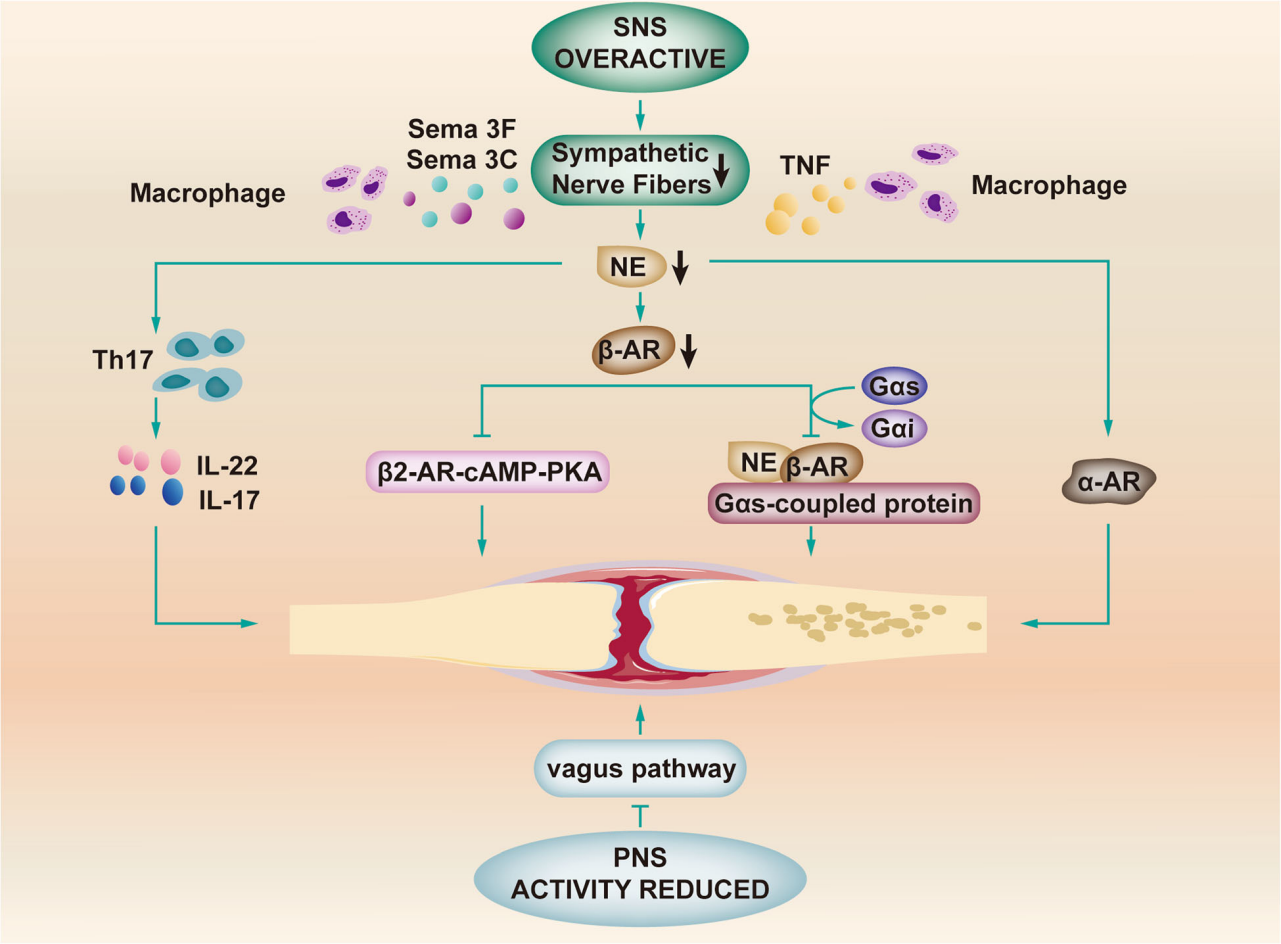
Relevant mechanisms of RA In RA, reduced PNS activity can inhibit the vagus pathway, contributing to the formation of inflammatory lesions. The overactivated SNS, as well as sema3C, sema3F, and TNF secreted by macrophages play important roles in the reduction in sympathetic nerve fibers. Then, the concentration of NE is decreased. Decreased NE promotes the secretion of IL-22 and IL-17 by Th17 cells. In addition, NE can activate α-AR and reduce the expression of β-AR. The latter inhibits the β2-AR-cAMP-PKA pathway and suppresses NE and β-AR binding to Gαs-coupled proteins by transforming Gαs to Gαi. All these processes are beneficial to the formation and persistence of inflammation in RA

The receptors of Sema3C and Sema3F are present on sympathetic nerve endings in the periphery. Their binding to their receptors leads to sympathetic nerve fiber repulsion in the neurite outgrowth assay [103]. TNF is an important inflammatory mediator. A study of Parkinson’s disease demonstrated that overexpressed TNF can reduce the expression of tyrosine hydroxylase (TH) and that TNF is toxic to catecholaminergic neurons. Moreover, TNF inhibitors can downregulate various cytokines in a murine collagen type II-induced arthritis model [131]. Hence, a high level of TNF and the existence of nerve repellent factors of sympathetic nerve fibers might be the causes of the loss of sympathetic nerve and low concentration of NE (Fig. 5).

## 4. Possible relationship between the ANS and endometriosis-associated inflammation

**Fig. 6:**
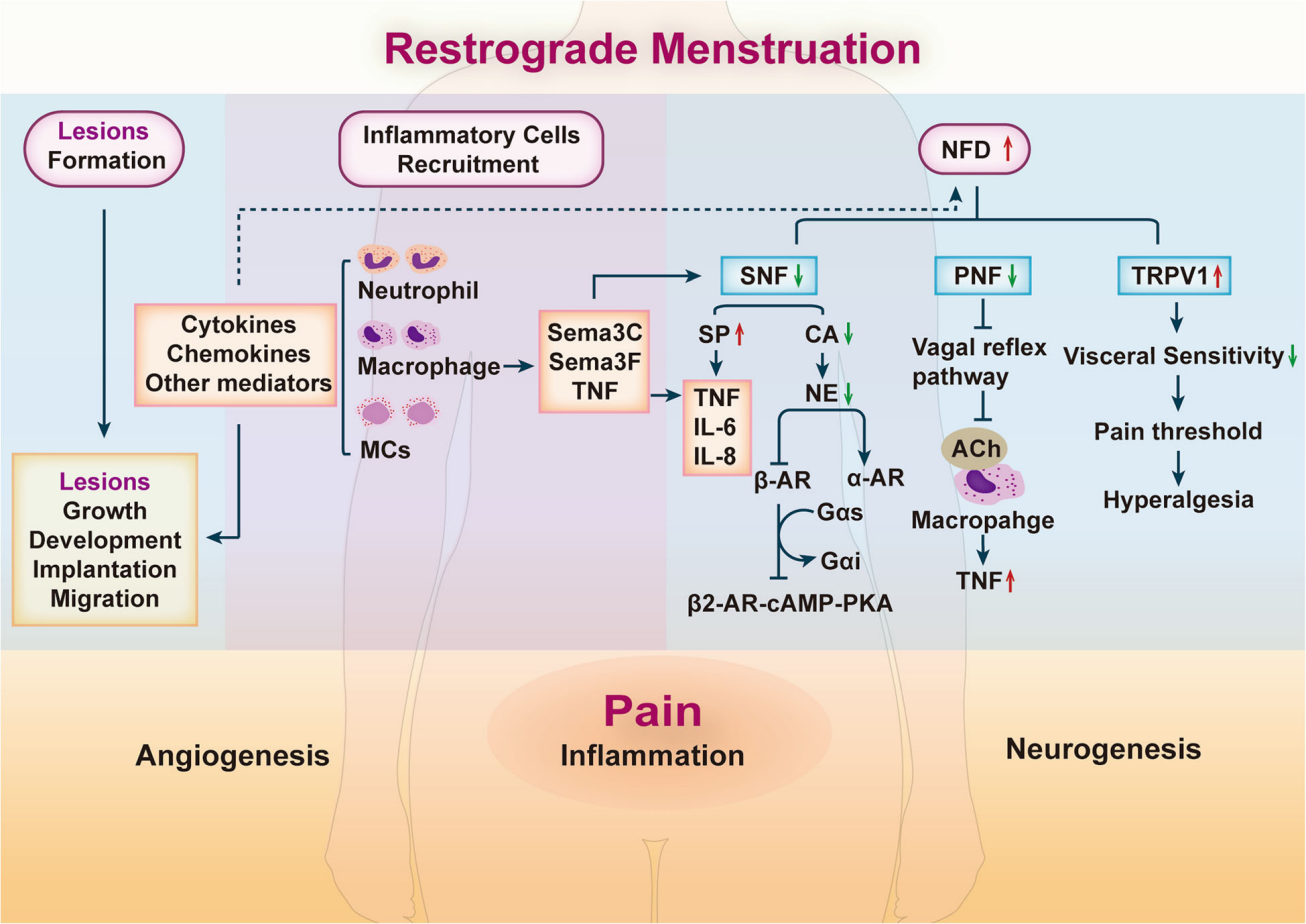
Possible relationship between the ANS and endometriosis-associated inflammation endometriotic lesions are formed after retrograde menstruation. Inflammatory cells (neutrophils, macrophages, MCs, and other cells) are recruited to endometriotic lesions, and total nerve fiber density (NFD) is increased. Sympathetic and parasympathetic nerve fiber densities are reduced in endometriotic lesions. Decreased SNF can induce an enhancement of SP-positive nerve fibers and a decreased in CA concentrations. Sema3C, Sema3F, and TNF secreted by macrophages also play vital roles in the reduction in SNF. The binding between ACh and the α-7-nicotinic ACh receptor of macrophages is restrained when the vagal reflex pathway is suppressed, causing an increase in TNF. The three main parts of endometriotic lesions cause the formation and persistence of inflammation, angiogenesis and neurogenesis. Ultimately, most women with endometriosis have various pain symptoms

As mentioned above, endometriosis has much in common with RA and IBD-chronic inflammation conditions, changes in inflammatory mediators, and the distribution of nerve fibers. The latter has been demonstrated in previous studies. However, it is not clear how the aberrant sympathetic and parasympathetic innervation in endometriosis is involved in the inflammatory reaction. Only a few studies support this phenomenon. In RA and IBD (especially in CD), relative mechanisms have been confirmed. According to these studies, we propose the possible relationship between the ANS and endometriosis-associated inflammation (Fig. 6).

### How does inflammation cause abnormal distribution and function of the ANS?

Multiple inflammatory cells (such as macrophages, neutrophils, and MCs) are recruited to the lesion after the initiation of endometriosis, due to the inflammatory processes activated by the endometrial debris [24]. After that, neutrophils and MCs, various cytokines (TNF-α, TGF-β, IL-1β, IL-6, IL-8, IL-37, etc.), chemokines (MCP1, CXCL8, CCL2, CCL5, etc.), and other inflammatory mediators (BDNF, NGF, etc.) are secreted [5, 7, 8, 10, 14–17].

Among these cytokines, IL-1β promotes the production of BDNF through an IL-1 receptor-dependent fashion, mediated by JNK, NF-κB, and the rapamycin signal transduction pathway [5, 67, 96]. The increased BDNF colocalizes with macrophages and nerve fibers, and can promote the growth, survival, and differentiation of several types of neurons [5, 68]. These factors interact with each other and participate in the development of inflammation as well as the formation of damaging pain in the peripheral and central nervous systems [94].

NGF is secreted by inflammatory cells and upregulated by cytokines (such as TNF-α and IL-1β) [5, 7, 10, 70]. The expression of NGF and its receptor TrkA is significantly enhanced in endometriotic epithelium and stroma [3, 22, 93]. NGF-TrkA signaling is involved in chronic pain and neuroinflammation [92]. NGF gives rise to the expression of SP and calcitonin gene-related peptide, contributing to the sprouting of nociceptors and increased number of sensory neurons [6, 65]. Under the influences of BDNF and NGF mediated by cytokines, total NFD is elevated. Finally, increased NGF and BDNF lead to persistent inflammatory pain.

Macrophages produce nerve repellent factors (Sema3C and Sema3F, etc.) specific for sympathetic nerve fibers [2, 11]. In addition, sympathetic nerve endings in the periphery have the receptors of Sema3C and Sema3F [103]. The binding to their specific receptors leads to sympathetic nerve fiber repulsion in the neurite outgrowth assay. Increased TNF-α secreted from inflammatory cells affects the early stage of endometriosis via activating local or systemic inflammation, and also has toxic effects on CA neurons [131]. Overexpression of Sema3C, Sema3F, and TNF will induce the loss of sympathetic nerve fibers in endometriosis.

In addition, we propose that abnormal secretions of cytokines, chemokines, and other mediators by the dysfunction of macrophage and MCs form a vicious cycle in endometriosis lesions. This cycle may aggravate the inflammatory neuropathic pain in endometriosis.

### How does dysfunction of the ANS cause inflammation?

The loss of sympathetic nerve fibers is a marker of the transition from acute to chronic inflammation [102–104]. This process leads to a reduction in the concentration of CA, while the concentration of SP is elevated. Pro-inflammatory peptide SP can induce increased secretion of pro-inflammatory cytokines (such as TNF, IL-6, and IL-8) [10, 11, 65, 102]. In addition, CA sometimes can act as an anti-inflammatory neurotransmitter. Therefore, the reduction of CA and increase of SP can weaken the anti-inflammatory effect and enhance the pro-inflammatory effect of endometriotic lesions, thus maintaining the inflammatory microenvironment as well as sustaining the development of inflammatory lesions.

The reduction of CA (the main component of reduction is NE) activates α-AR while inhibits β2-AR [132, 134, 135]. Normally, NE binds to the β2-AR and then transmits information via Gαs-coupled proteins [103]. However, the inflammatory environment can disrupt this anti-inflammatory pathway. At the same time, the Gαs-pathway (high cyclic AMP) of receptor coupling can switch to the Gαi-pathway (low cyclic AMP), which is a pro-inflammatory signal [103]. Therefore, the low concentration of NE and downregulation of β2-AR possibly lead to a pro-inflammatory response in lesions. It can be assumed that the reduced concentration of NE will lead to an enhancement of inflammation in endometriotic lesions through the aforementioned mechanisms.

However, CA does not always act as an anti-inflammatory neurotransmitter. It can also act as proinflammatory or in a dose/time-dependent biphasic manner. Depressed catechol-O-methyltransferase (an enzyme that metabolizes catecholamines) can increase the levels of NE and epinephrine, which activate β2/3ARs to produce heightened pain sensitivity [136]. Instead, NE can activate β-AR to reduce LPS-induced inflammatory responses in bone marrow-derived macrophages. After sympathectomy, mRNA expression levels of pro-inflammatory cytokines are increased and mRNA expression level of anti-inflammatory cytokine IL-10 is decreased, suggesting that NE can play an anti-inflammatory role in this situation [101]. Härle P et al. have demonstrated that the SNS has time-dependent opposing effects on the severity of CIA. The SNS has pro-inflammatory effects in early stage and confers anti-inflammatory effects in the later stage [137]. The bimodal effect of the SNS is dependent on the time point of immune system activation and the respective compartment [137].

The reduction in parasympathetic NFD and inhibition of the vagal reflex pathway alter the brain-gut axis [117, 118]. After the transmission of changes in the intestinal microenvironment from gut to CNS, an inappropriate response occurs. This response helps to maintain the pathological state of the intestine and promotes the development of disease. In addition, the concentration of ACh, released by synapses of enteric neurons, is decreased. As a result, the binding between ACh and the α-7-nicotinic ACh receptor of macrophages is restrained. This process leads to increased expression of cytokines such as TNF, maintaining persistent intestinal inflammation [115, 128]. In addition, inflammatory mediators can stimulate TRPV1 and other peripheral nociceptors [129]. TRPV1-positive nerves induce the secretion of neurotransmitters from abnormal nerve fibers of endometriotic lesion. This process can in turn aggravate the local neurogenic inflammatory response.

The above changes in ANS can inhibit the anti-inflammatory pathway and enhance the effect of the pro-inflammatory effect. Afterwards, the production of pro-inflammatory mediators is elevated, leading to inappropriate responses. These factors maintain the inflammatory pathological state of endometriotic lesions.

### How does the interaction between inflammation and the ANS participates in endometriosis?

The possible relationship between inflammation and ANS has been mentioned above. In brief, inflammatory cells promote the production of cytokines, chemokines, and other noninflammatory factors. Elevated levels of neurotrophins lead to an increase in total NFD. Moreover, the sympathetic nerve repellent factors Sema3C and Sema3F also secreted, leading to sympathetic nerve fiber repulsion. As a result, the expression of SP is increased. These factors in turn promote the release of inflammatory mediators. Moreover, enhanced activation of NF-κB contributes to the elevated expression of inflammatory factors. This interaction of inflammation and the ANS promotes the development of inflammation in endometriotic lesions and has a significant impact on the occurrence and development of lesions and pain.

Thus, how does this relationship participate in the development of endometriosis? Additionally, how does this interaction cause pain and participate in the development of pain?

Angiogenesis and cell proliferation are vital to the development of endometriotic lesions. The immune-predominant phase and hormone-predominant phase contribute to the development and maintenance of endometriosis. Angiogenesis belongs to the initiation phase (< 72 h after disease initiation). After implantation of ectopic endometriotic lesions, a new vascular system is needed to maintain the survival and sustained growth of ectopic lesions. Angiogenesis is beneficial to the survival, growth, implantation, and migration of endometriotic lesions [17, 23, 24, 31, 32, 56–58]. In cancer, activated macrophages can increase the migration and invasion of tumor cells as well as anti-tumor immunity [24, 26]. Analogously, in endometriosis, the recruitment of macrophages into endometriotic lesions leads to the production of PGE2/TGF-β, resulting in the inhibition of the immune response [3, 10, 20, 22]. Macrophages can also co-occur with the recruitment of circulating endothelial progenitors and sustain their survival as well as the integrity of the vessel wall [24]. The hypoxic environment of local endometriotic lesions stimulates overexpression of COX-2, resulting in aberrant production of PGE2. PGE2 stimulates steroidogenesis, angiogenesis, and immunologic suppression in endometriosis. Moreover, hypoxic conditions promote angiogenesis through IL-8 from macrophages, contributing to the elevated migration of ESCs [4–6, 58]. Increased macrophages and neutrophils enhance the expression of VEGF and promote angiogenesis [42, 44]. In addition, the development and distribution of vessels and nerve fibers are synchronous in the rat model of endometriosis and they are always concomitant [3]. Abundant angiogenesis also provides conditions for the growth of nerve endings. Both the vessels and nerves affect the development of endometriotic lesions. The abundant blood supply of neovascularization provides adequate nutrition for the growth of lesions and nerve endings, contributing to adhesion, invasion, and aberrant innervation of endometriosis.

Multiple inflammatory and noninflammatory mediators are essential for the development of endometriotic lesions. IL-10 contributes to proliferation and implantation of ectopic lesions. IL-8 is associated with cell proliferation and growth of endometrial cells and has chemotactic effects on neutrophils, resulting in the transformation of acute inflammation to chronic inflammation [17, 56–58]. The continuous activation of MCs and the production of IL-4 and IL-6 are related to the production and maintenance of persistent inflammation, leading to deterioration of endometriosis [19, 61]. NGF is involved not only in the growth of neonatal nerve fibers but also in the activation of MC degranulation. The occurrence and maintenance of persistent inflammation are related to sustained activation of MCs and the production of IL-4 and IL-6 [3, 5, 7, 10]. As a result, these effects contribute to the exacerbation of endometriosis. In addition to the involvement of neurogenesis, NGF can activate MC degranulation to produce more inflammation-associated mediators, such as PG [70]. PG activates NF-κB to promote the expression of inflammation-related genes [67]. Then chemokines are increased, and macrophages and neutrophils are recruited in endometriotic lesions, leading to the enhancement of chronic inflammation. RHOGTPases (related to cell migration and adhesion) activate P38MAPK through TGF-β, resulting in increased angiogenesis, development of endometriotic lesions, and aggravation of inflammation [63, 64]. In addition, TNF-α has a toxic effect on the sympathetic nerve [131], leading to decreased secretion of anti-inflammatory mediators and increased production of pro-inflammatory mediators. All of these factors contribute to endometriosis progression and deterioration. The recruitment of neutrophils causes increased secretion of IL-17, leading to the proliferation of ESCs [16, 47, 60]. This process promotes cell adhesion. Cell adhesion impels the enhanced expression of TNF-β1, IL-1, and TNF-α via the P38/ERK1/2 signaling pathway. This process contributes to the expression of IL-8 and VEGF [58]. These effects in turn promote the proliferation of ESCs and adhesion of vascular cells.

Changes of microenvironment and ANS in endometriosis participate in the mechanism of endometriosis-associated pain. Many aspects of inflammatory microenvironment can induce pain. Extensive activation of MCs causes more secretion of NGF, resulting in increased growth of nerve fibers. Next, nociceptors are activated, and peripheral nociceptive endings are sensitized. Ectopic implantation of the endometrium can release molecular signals that act on peripheral nociceptive endings, depolarizing them and generating AP. These effects can reduce the pain threshold so that harmless stimuli can activate high-threshold nociceptors. Moreover, NGF promotes sprouting of nociceptors and increases the number of sensory neurons, contributing to persistent chronic inflammatory pain. In addition, activated MCs release histamine, interleukin, TNF-α, and PGs, causing neuropathic pain. The inflammatory factor IL-1β also reduces the mechanical nociceptor threshold and activates the nociceptive nerve. BDNF not only participates in endometriosis-associated inflammation but also promotes peripheral and central nervous systemic pain. BDNF plays a role in the relationship between inflammatory pain and neuropathic pain via some signaling pathway. During the inflammatory state, nerves carry damage signals to the brain, then the sensitized CNS release peptides into the local environment. Once C sensory nerve fibers transmitting the pain signal are activated, even if inflammation has been eliminated, continuous electrical activity and pain still exist. Increased TRPV1 in endometriotic lesions can regulate the generation of AP and induce persistent AP, leading to a change in visceral sensitivity and reduction in the threshold of pain. TRPV1-positive nerves trigger the release of neurotransmitters from peripheral tissues and enhance local neurogenic inflammation. Both promote the development of inflammation, produce hyperalgesia, and eventually produce endometriosis-associated pain.

## 5. The influence of sex hormone on inflammation and ANS contribute to endometriosis

It is known that endometriosis is an estrogen-dependent disease [1]. Compared with healthy women, local levels of estrogen are higher in menstrual blood and PF of patients with endometriosis [138]. As mentioned above, the imbalance of immune cells and abnormal innervation of nerve fibers (sympathetic, parasympathetic, and sensory nerve fibers) are proved in endometriotic lesions. So, whether estrogen is involved in endometriosis-associated inflammation and ANS changes?

Estrogen can regulate the recruitment of immune cells via ERα and ERβ in different ways, contributing to an abnormal inflammatory environment in endometriosis. Erin Greaves et al. found that all endometriotic lesion-resident macrophages (in human tissues and mouse model of endometriosis) expressed ERβ and up to 20% macrophages expressed ERα [139]. Another in vitro experiment has demonstrated that ERβ can elevate CCL2 through NF-κB in ESCs, and increased production of CCL2 leads to ERβ-mediated macrophage infiltration [140]. Furthermore, we also reported that increased estrogen could recruit MCs and macrophages to endometriotic lesions in many ways [78].

Besides inflammation, there are evidences that estrogen also affects the change of nerve fibers. In murine uterus, estrogen can inhibit sympathetic neurite outgrowth through regulating BDNF synthesis [141]. Acute administration of 17β-E2 to adult ovariectomy rates lead to an 85% reduction in the density of myometrial sympathetic nerves at 24 h [75]. In addition, other studies have demonstrated that sympathetic nerves are reduced in myometrium of women with estrogen-dependent disease adenomyosis [142]. The sympathetic denervation of rat myometrium is correlated with estrogen-induced collagen reorientation [143]. Long-term exposure to estradiol-17β leads to decreased cholinergic innervation in pig ovary [144]. Cyclic variations of sex hormone strongly influence uterine innervation, especially sympathetic nerves. The level of NA is also affected by this fluctuation [75]. In murine uterus, E2 regulates the synthesis of BDNF (exert a repulsive effect on sympathetic neurites through p75^NTR^) to influence neurogenic properties. BDNF activates sphingomyelinase and increases intracellular ceramides level through p75^NTR^ receptors presented on sympathetic axons [75]. This process in turn impedes sympathetic outgrowth [75]. NGF also plays a role in the change of nerve fibers in endometriosis. It is essential for the development and survival of adrenergic and sensory neurons both in CNS and peripheral nervous systems. TrkA and p75^NTR^ are required for carrying out the modulatory effects of sympathetic neurons of NGF. Interestingly, the survival of sympathetic neurons loss dependence on NGF, but axonal growth of sympathetic neurons remains under the influence of NGF. In high concentration of E2 environment, increased NGF prefers to promote axonal growth of sensory nerve fibers, and inhibit mature sympathetic ganglia outgrowth [75, 78]. As a consequence, estrogen is important in the regulation of abnormal innervation in female reproductive system.

According to the evidence discussed above, we proposed that estrogen can influence the interaction of inflammation and nerve fibers in endometriosis. In our previous study [145], we have reported that increased E2 could activate MCs to secrete NGF leading to sensitize dorsal root ganglion cells. After peripheral axon injury, neuron releases exosomal miR-21-5p mediated by estrogen signaling pathway. After that, macrophage phagocytizes miR-21-5p, which contributes to the infiltration of macrophages toward peripheral nerves. At the same time, peripheral nerves also secrete CSF-1 and CCL2 to enhance macrophages migration to endometriotic lesions [145]. In an animal experiment, rat DRG, treated with E2, can increase mRNA level of CCL2, which stimulates the migration of CSF-1-differentiated macrophages. In turn, macrophages treated with E2 can elevate concentrations of BDNF and NT3, promoting neurite outgrowth from ganglia explants [139]. According to these evidences, we suggest that estrogen not only influences the number and function of immune cells but also mediates the change of ANS in endometriotic lesions. More importantly, estrogen can influence the interaction of inflammation and nerve fibers in endometriosis.

As mentioned above, inflammation, or changes of ANS and sensory nerve fibers, can cause the development of endometriotic lesions and promote endometriosis-associated pain. According to statistics, 60% of women with painful periods suffer from endometriosis. Dysmenorrhea, pelvic pain, dyspareunia, and dyschezia are the most common pain symptoms of endometriosis, which seriously affect women’s quality of life, career, and daily activities [146, 147]. In order to improve the quality of life of women with endometriosis, several drugs are commonly used in clinic to relieve pain and inhibit the development of lesions.

First-line and second-line medicine currently available for endometriosis-associated pain include nonsteroidal anti-inflammatory drugs (NSAIDs), hormone therapy, and gonadotropin-releasing hormone (GnRH) agonists and antagonists [148]. NSAIDs are the most commonly used first-line medicine. It works by blocking the COX enzyme, which is important for the production of inflammatory factors [146]. However, if used for a long time, the risk of hypoestrogenic adverse effects, negative calcium balance, and osteoporosis increases [149].

GnRH antagonist is a type of prevalent medicine for endometriosis. GnRH antagonist downregulates the release of follicle stimulating hormone and luteinizing hormone via blocking the GnRH receptor in pituitary cells, leading to the suppression of ovulation [145]. GnRH agonists and antagonists are effective in the relief of endometriosis-associated pain, but they are also associated with tolerable hypoestrogenic adverse effects (vasomotor symptoms, genital hypotrophy, and mood instability) and negative calcium balance with an increased risk of osteopenia [149, 150]. Hormonal drugs can relieve pain in more than 90% of endometriosis patients [151]. These drugs inhibit ovulation and menstruation and have similar beneficial effects against pain. However, only estrogen-progestins and progestins have safety, tolerability, and cost profiles that allow long-term use [150].

Dienogest (DNG), to be marketed in mainland China, is a steroidal fourth-generation selective progestin that combines the pharmacologic properties of 19-nortestosterone, a derivative of progesterone. DNG combines potent progestogenic efficacy and moderate estrogen-suppressive effects to effectively reduce the growth of endometrial-like tissue, combined with anti-inflammatory, antiproliferative, and antiangiogenic effects. This treatment modulates the production and metabolism of PGs in a way that is anti-inflammatory. Moreover, the use of DNG is associated with proinflammatory cytokine and chemokine production, as well as growth factor biosynthesis and signal kinases, which are responsible for the control of inflammation. Although DNG produces other progesterone-like side effects (e.g., weight gain, increased blood pressure, breast tenderness, and nausea), it has no androgenic side effects and has little effect on metabolic and lipid parameters. As a result, DNG has been shown to be well tolerated [152, 153].

In addition to inflammatory mediators mentioned above, a laboratory-based study found that use of hormonal therapy is associated with reduced NFD in endometriosis (especially in DIE) [154]. Progesterone can increase vaginal sympathetic (but not parasympathetic) nerve terminals in female Sprague-Dawley rats, whereas E2 can reduce innervation in progesterone-treated rat and untreated rat [155]. These effects of progesterone in nerve fibers may also relieve pain in women with endometriosis who use progesterone.

## 6. Potential implication of ANS and inflammation interaction in the management of endometriosis treatment

The treatment objectives of this chronic inflammatory disease are to relieve endometriosis-associated chronic pain, and to achieve pregnancy successfully in infertile women [156]. There are many methods for treating endometriosis, including medical treatment and surgical treatment [146]. However, the current treatment methods are not as effective as expected. Although pain can be managed through pharmacological inhibition of ovulation and menstruation, medical therapy is symptomatic not cytoreductive. In addition, surgery is usually associated with pain relief, but its effect is always temporary [149].

The effects of surgery on pain are often temporarily satisfactory. Peritoneal implants can be safely coagulated or excised with similar benefits. According to the progress of endometriosis or requirements in ovarian endometriosis patients, conservative ovarian surgery or excision of ovarian endometriomas is selected. For DIE patients, excisional surgery is the best way for women with symptomatic DIE especially intestinal endometriosis. Good pain relief is generally reported during the first year after bowel resection for DIE [149, 157].

In addition to the main drugs for endometriosis mentioned in the previous section (part 5 “The Influence of Sex Hormone on Inflammation and ANS Contribute to Endometriosis”), there are other medicines that can relief pain through affecting inflammatory response. Raloxifene can decrease M1 monocytes, macrophage density, and the NF-κB response associated with a decrease in NO, IL-1β, and IL-6 production. This property can make raloxifene effective in the treatment of chronic pelvic pain for endometriotic patients [145]. Pesveratrol is a new type of natural phenolic drug for the prevention and treatment of endometriosis. The basic mechanism of effect is considered to be anti-inflammatory reactions. Pesveratrol can inhibit the synthesis of PGs through inhibiting COX enzyme synthesis and activating immune cells and pro-inflammatory cytokines [156].

In view of the adverse reactions of the above drugs, there is an urgent need for innovative and more effective treatment methods. Selective estrogen receptor modulators provide a novel treatment strategy for endometriosis, causing both agonistic and antagonistic activity at the ER [145]. Glucosaminyl muramyl dipeptide prevents hyperactivation of macrophages to weaken inflammation in lesions [26]. In addition, antagonizing the EP4 receptor signal not only inhibits the proliferation of endometrial cells induced by PGE2 but also regulates anti-inflammatory activity and alleviates inflammatory pain. Moreover, this receptor is present in sensory nociceptive nerve fibers. Pain can enhance the expression of the EP4 receptor in dorsal root ganglion neurons. Therefore, human PGE2 receptor subtype 4 antagonists may be an effective new drug in the treatment of endometriosis [157].

Considering the similar mechanism among IBD, RA, and endometriosis described in the above section, we hope to find some new and more effective methods for endometriosis by referring to the treatment methods of these disorders. Infliximab is a TNF-α inhibitor. The clinical effect of infliximab is good in RA, although it has high costs [158]. Applying infliximab to treatment of endometriosis may not only inhibit TNF to control inflammation but also reduce the toxic effect of TNF on sympathetic nerve fibers. However, a review of drug development of endometriosis has described that this drug failed midway during development [159]. Hyaluronic acid (HA) can bind to the cell surface adhesion molecule CD44. CD44 is overexpressed in activated macrophages and synoviocytes under RA conditions. The hyaluronic acid-methotrexate conjugate can release methotrexate in inflamed tissue, resulting in downregulation of inflammatory cytokine levels and a reduction in cartilage damage in arthritic mice [158]. The concentration of soluble CD44 in the serum and PF of endometriotic patients is higher than that of healthy women [160, 161]. According to this mechanism of hyaluronic acid-methotrexate, is it possible to consider designing a drug with a similar structure for endometriosis? Peptidyl arginine deiminase 4 (PAD4), an initiator of citrullination, is an important therapeutic target for inflammatory diseases. In-depth studies have been carried out on tumors and multiple inflammatory disorders (such as RA). The expression of PAD4 is regulated by an ERα-dependent mechanism. Estrogen can regulate the expression of PAD4 via classical and nonclassical pathways at the transcriptional level. Moreover, PAD2 is expressed in the female genital tract and regulated by estrogen and epidermal growth factor in reproductive tissues [162]. As a result, PADs may be a new therapeutic target for endometriosis.

In neuropathways, vagal nerve stimulation can reduce serum IL-6 concentrations significantly in RA. The level of reduction is related to the degree of RA improvement [163]. This treatment method may lessen concentrations of IL-6 and other cytokines, which can weaken inflammation in endometriotic lesions. In terms of immune homeostasis, great progress has been made in the study of dendritic cell (DC) therapeutics in RA mouse models. In CIA models, the use of low doses of semimature DCs can suppress disease progression by elevating the Treg population and inhibiting antigen-specific Th1- and Th7-mediated immunity. Treatment of CIA mice with tolerogenic DCs modified by tacrolimus significantly inhibited the severity and progression of disease by altering the proportion of Th1 and Th17 cells in the spleen [163]. The development of endometriosis is dependent on the presence of endogenous DCs. Mannose receptors on peritoneal DCs can enhance the phagocytosis of dead ESCs. DCs secrete IL-1β and IL-6 promoting the escape of immune surveillance and Th17 differentiation. Moreover, markedly decreased DCs cause larger endometriosis-like lesions and significantly reduce the activation of T lymphocytes [164]. In brief, the methods of DC therapeutics may be used for reference in endometriosis treatment.

However, there is still an urgent need to identify new targets and to further develop new drugs for endometriosis treatment to control the development of endometriosis and improve the quality of life of endometriotic patients.

## Conclusion

Endometriosis is a complex, estrogen-dependent, and chronic inflammatory gynecological disease, associated with infertility and endometriosis-associated pain [26, 99]. Although the mechanisms require clarification, according to current studies, many researchers believe that inflammation and the changes of nerves in endometriotic lesions are related to the occurrence and development of endometriosis. The interaction between inflammation and nerves can aggravate endometriosis and is closely correlated with endometriosis-associated pain. Inflammation is considered to be a significant cause of pain because of the neurotropic and neuroprotective activities of cytokines [72]. In addition, abnormal innervation leads to changes in neurotransmitters and aberrant secretion of inflammatory factors. These changes can mediate neurogenesis and subsequent peripheral neuroinflammation in endometriosis [26].

The rich ground plexus of autonomic nerves is widely distributed in the female reproductive system, regulating glandular secretion, immune cell interactions, etc. This plexus also conveys information to the CNS regarding the internal environment and potential noxious stimuli [75]. Changes in the systemic levels of estrogen can regulate the remodeling of the uterine sympathetic nerve in nonpregnant females and promote the secretion of chemokines from peripheral nerves [75, 76, 145]. Due to the alterations of sympathetic and parasympathetic innervation and function in endometriotic lesions, the effects of anti-inflammation of the sympathetic and vagal reflex pathways are restrained, contributing to the formation and development of the pro-inflammatory microenvironment. Although certain mechanisms of endometriosis are still unclear, we can draw on the experience of studies of IBD, RA, and other chronic inflammatory disorders to determine a breakthrough aspect for further endometriosis studies and to provide some clues for new therapeutic targets.

## Data Availability

The data supporting the conclusion of this article is included within the “References” section.

## Abbreviations

Ach: Acetylcholine
AIDs: Inflammatory autoimmune diseases
ANS: Autonomic nervous system
AR: Adrenergic receptor
BDNF: Brain-derived neurotrophic factor
CA: Catecholamine
CD: Crohn’s disease
CHUK: Conserved helix-loop-helix ubiquitous kinase
CIA: Collagen-induced arthritis
CNS: Central nervous system
DCs: Dendritic cells
DIE: Deep infiltrating endometriosis
DNG: Dienogest
DRG: Dorsal root ganglia
E2: Estradiol
ER: Estrogen receptor
ESCs: Endometrial stromal cells
GnRH: Gonadotropin-releasing hormone
Gαi: Galphai
Gαs: GalphaS
IBD: Inflammatory bowel disease
IFN: Interferon
IL: Interleukin
JNK: c-Jun N-terminal kinase
LPMs: Large peritoneal macrophages
MCs: Mast cells
MMs: Muscularis macrophages
NFD: Nerve fiber density
NFKBIA: NF-κB inhibitor alpha
NGF: Nerve growth factor
PAD: Peptidyl arginine deiminase
PF: Peritoneal fluid
PGs: Prostaglandins
PNFs: Parasympathetic nerve fibers
PNS: Parasympathetic nervous system
RA: Rheumatoid arthritis
SNFs: Sympathetic nerve fibers
SNS: Sympathetic nervous system
SP: Neuropeptide substance P
SPMs: Small peritoneal macrophages
TGF-β: Transforming growth factor-β
TH: Tyrosine hydroxylase
TNF: Tumor necrosis factor
TRPV1: Transient receptor potential cation channel subfamily V member 1
UC: Ulcerative colitis
VAChT: Vesicular acetylcholine transporter
VEGF: Vascular endothelial growth factor
